# Simultaneous determination of gross alpha/beta activities in water by liquid scintillation counting and its applications in the environmental monitoring

**DOI:** 10.1038/s41598-022-12245-x

**Published:** 2022-05-18

**Authors:** Xiaoyun Li, Shaolin Wang, Hailin Lou, Jingshun Pan, Qian Dong, Yifan Zheng, Ling Chen

**Affiliations:** China Institute of Atomic Energy, Beijing, 102413 China

**Keywords:** Environmental monitoring, Environmental monitoring, Natural hazards

## Abstract

Based on the standards of ISO11704-2018 and ASTM D7283-17, a method for simultaneous determination of gross alpha and gross beta activity concentrations in water by liquid scintillation counting (LSC) was established, which can be applied to various types of water samples in routine monitoring, such as drinking water, groundwater, geothermal water, seawater, and radioactive wastewater. The sample’s pH value and concentrated volume must be controlled to avoid quenching as much as possible. The validation tests show that the deviations of gross alpha and gross beta activities can satisfy quality control requirements in a wide range of activity ratios from 1:102 to 67:1. For the actual samples, the measurement results of the LSC method are in good agreement with those of the thick source method, in which the relative deviations of gross alpha and gross beta are both less than 15% for these two methods. Moreover, the LSC method performs better in detection limit and has a simpler pretreatment process than the thick source method.

## Introduction

Radioactivity is widely present in various kinds of water bodies, such as seawater, river, drinking water, groundwater, and wastewater. The measurements of gross alpha and gross beta activity concentrations are among the most effective methods for preliminary screening and evaluating the total radioactivity contents in samples. In China, the national standard for drinking water quality stipulates that the guidance values of gross alpha and gross beta are 0.5 Bq L^−1^ and 1 Bq L^−1^, respectively^1^, which is the same as that of the WHO guideline^2^. For wastewater, the maximum allowable discharge concentrations of gross alpha and gross beta are 1 Bq L^−1^ and 10 Bq L^−1^, respectively^3^. Once the radioactivity index exceeds the limit values, nuclide analysis and evaluation should be carried out targeted. So it is essential to establish a sensitive, rapid, and simple method to measure gross alpha and gross beta activities in the water for routine monitoring of many samples. The gross alpha and gross beta activities in water can be measured by gas-flow proportional counting (or ZnS(Ag) solid scintillation counting) and liquid scintillation counting (LSC)^4–7^. Compared with the former, the latter can obtain a lower detection limit in a shorter time because of its 4π detection efficiency and simple operation^8–10^.

A pulse shape analysis (PSA) equipped with LSC is used to discriminate α/β events, in which alpha and beta counts are stored in separate storage addresses of the multichannel analyzer (MCA). The PSA value can be set from 1 to 256, and the optimal one needs to be chosen by measuring a pure alpha emitter solution and a pure beta emitter solution. Lower PSA value being set will cause more β-pulses to fall into the α-MCA, and a higher PSA value will cause more α-pulses to fall into the β-MCA. This misclassification can be minimized by using the optimized PSA value. Quenching must be considered in liquid scintillation measurement. Different degrees of quenching will change the optimal PSA value and affect the detection efficiency and the separation effect of α/β. Researchers have deeply studied the quenching of LSC measurement. The factors causing quenching include the acid type and intensity^12,13^, total dissolved solids (TDS)^10,14,15^, and color of samples^16–18^. It is generally required to add nitric acid when environmental water samples are collected. However, the increase in acidity will lead to the rise of quenching. An alternative method is to concentrate samples under the condition of controlling the pH value to achieve the same quenching level^6,19,20^. Different types of water samples have different contents of soluble solids. The amount of dissolved solids in the sample is generally controlled to be no more than 400 mg to ensure the formation of a homogeneous solution after mixing with a cocktail^7^. When the sample has color, a color quenching correction is required^16^.

There are different quenching correction methods according to the types of detectors for the commercial liquid scintillation analyzers. In the case of the conventional LSC, two photomultiplier tubes (PMTs) are faced the vial and positioned at 180° relative to each other to detect the light emitted from the vial. And an external γ-source (^137^Cs, ^226^Ra, ^152^Eu, or ^133^Ba) is equipped to determine the quench by measuring the shift of the Compton spectrum obtained from the external standard^21,22^. Another newly developed method is called the triple-to-double coincidence ratio (TDCR) efficiency calculation technique. The TDCR method requires three PMTs at 120° each other, and to obtain dual and triple phototube coincidence outputs. The triple and double coincidence counting rates are measured and the ratio of these coincidences is calculated. The change of counting efficiency due to quenching of the sample will manifest itself in the measured triple and double counting rates, thereby producing a different TDCR value. Therefore, quenching correction can be carried out by establishing the relationship between TDCR value and detection efficiency. The TDCR method was originally used for radionuclide standardization. Until 2008, the liquid scintillation analyzer with three PMTs can be commercially available, which makes the TDCR method gradually applied in the field of radioactivity monitoring^21,23–26^. In the paper, the quenching levels of samples are detected via an external γ-standard.

In China, the approved methods for measuring gross alpha and gross beta activities in the water are the thick source methods. The purpose of this paper is to establish a method for the determination of alpha and beta activities in water by LSC based on ISO11704-2018^6^ and ASTM D7283-17^7^ standards. And suitable pretreatment conditions are experimented. By controlling the sample acidity and concentration ratio, etc., the quenching level of the sample is controlled, so that the method can be used in routine monitoring for different types of water. For the actual water samples, the reliability and applicability of the LSC method are further investigated by comparing the results of the thick source method.

## Materials and methods

### Apparatus

An ultra-low background liquid scintillation spectrometer Wallac 1220 Quantulus manufactured by PerkinElmer (Finland, 2002) has been used for the measurements. A PSA is provided to discriminate α from β radiations and an external standard source of ^152^Eu to obtain external quench parameter SQP(E) for indicating the quench levels of samples.

A BH1227 4-channel low background α/β measuring assembly equipped with ZnS(Ag) solid scintillation counters (China Nuclear Control System Engineering Co. Ltd) was used to measure the conventional thick source method.

A Lei-ci PHS-3G pH-meter and a Lei-ci DDSJ-308F conductivity meter (Shanghai INESA Scientific Instrument Co. Ltd, China) were used for pH and TDS measurements, respectively.

A high pure germanium gamma spectrometry with a broad energy detector (BE6530, Canberra, USA) was used to measure gamma-ray emitting radionuclides in water. Its relative efficiency is 60%, and the energy resolution is 1.8 keV at 1332.5 keV of ^60^Co. The energy response range is from 15–3000 keV.

### Establishment of a method for the simultaneous measurement of gross alpha/beta activities in water by LSC

#### Reagents and materials

The pure ^241^Am and ^90^Sr/^90^Y standard solutions were used for LSC calibration. ^241^Am in 0.5 mol L^−1^ HNO_3_ (radio-purity > 99.9%) was provided by China Institute of Atomic Energy. ^90^Sr/^90^Y in 3 g L^−1^ HNO_3_ was purchased from Czech Metrology Institute. Both of them were diluted with 3 g L^−1^ HNO_3_ carrier solution. Then their activities were certified by the national first-class ionizing radiation metrology station with the values of 20.5 ± 0.2 Bq g^−1^ and 31.1 ± 0.3 Bq g^−1^ (with coverage factor *k* = 2 for 95% confidence), respectively. ^40^K standard solution was prepared using KCl (guaranteed reagent, purity > 99.8%) supplied by Macklin Biochemical Co. Ltd. (Shanghai, China). And the activity concentration of ^40^K was determined by using the ratio between natural and radioactive potassium. Cocktail Ultima Gold AB (PerkinElmer) and 20 mL polyethylene vials (PerkinElmer) were used for the LSC measurement.

Five solid salts of NaCl, MgCl_2_, CaCl_2_, Na_2_SO_4_, and NaHCO_3_ (analytical reagent) were obtained from Sinopharm Chemical Reagent Co. Ltd. (China). They were used to prepare saline water as a chemical quenching agent. Nitric acid (guaranteed reagent) was used to prepare carrier solution and acidify samples with received. All aqueous solutions were prepared with deionized water.

#### Calibration procedure of LSC

During LSC measurement, all samples and cocktail volumes were maintained at 5 mL and 15 mL, respectively. Sample activities were controlled by adding the quantity of the standard solution. The volumes of the final samples were made to 5 mL by adding nitric acid carrier solution (~ 3 g L^−1^, pH = 1.59 ± 0.03). All vials were placed inside the counter for at least two hours for dark adaptation before counting. For PSA optimization, 1 g of pure ^241^Am and ^90^Sr/^90^Y standard solutions were measured under different PSA settings for 10 min. And three replicates of each solution were performed. The blank sample consisted of 5 mL HNO_3_ carrier solution plus 15 mL of Ultima Gold AB was measured under the same conditions for 1000 min.

The most abundant ions in saline or seawater are Na^+^, Mg^2+^, Ca^2+^, Cl^-^, SO_4_^2-^ and HCO_3_^−^^27,28^. A simulated saline (SS) solution was prepared with five salts of NaCl, MgCl_2_, CaCl_2_, Na_2_SO_4_, and NaHCO_3_, referring to the ratio of the ions in seawater and saline published in the literature. The pH value of the SS solution was adjusted to about 1.6 with HNO_3_, and the specific contents of these salts are shown in Table 1. Using SS solution as a chemical quenching agent, 0.5 mL, 1 mL, 1.5 mL, 2 mL, and 3 mL was added to the ^241^Am and ^90^Sr/^90^Y standard solutions and measured under different PSA settings to investigate the quenching effect of TDS in the sample.

**Table 1:** Mass concentration of different salts in simulated saline water.

Salt	Concentration (g L^−1^)
NaCl	75.5
MgCl_2_	30.5
CaCl_2_	3.3
Na_2_SO_4_	10.2
NaHCO_3_	0.5
Total	120.0

A series of spiked samples with ^241^Am, ^90^Sr/^90^Y, and ^40^K were used as test samples to validate the calibration curve. They were measured under an optimal PSA condition for 300 min.

### Sample pretreatment for LSC measurement

Based on the sample procedures recommended in the standard of ISO11704-2018^6^, different pretreatment methods were adopted according to the types and characteristics of samples. For environmental water, such as drinking water, surface water, groundwater, and geothermal water, a thermal pre-concentration method was used. Different concentration ratios were adopted depending on the salt content of the sample. Generally, prior to pretreatment, the TDS value of the sample was measured to judge the appropriate concentration ratio of the sample preliminarily. Then a weighed aliquot of the water sample of approximately 200–500 g was taken into a beaker, acidified with a certain amount of HNO_3_, and slowly evaporated to a final quantity of roughly 10–20 g. The pH value of the concentrated aliquot was controlled at about 1.6. After cooled to room temperature, 5 mL of the concentrated aliquot was transferred into the vial and mixed with 15 mL of cocktail to obtain a homogeneous solution for LSC measurement. The measurement time was 300 min. The remaining solution in the beaker was dried completely, and the residue was weighed to calculate the exact mass of solid (*m*_*r*_, mg) in the sample of LSC measurement.

Samples with relatively high salt content such as seawater were directly measured after heating to remove the dissolved ^222^Rn. A weighed aliquot of the water sample of approximately 100 g was taken into a beaker, acidified with a certain amount of HNO_3_, and heated with a cover to around 80 °C while stirring for 30 min. The pH value of the aliquot was controlled at about 1.6. After cooled to room temperature, 5 mL of the aliquot was transferred into the vial and mixed with 15 mL of the cocktail. Then the remaining solution in the beaker was dried completely and weighed to obtain the solid residue quality of the sample. The exact mass, *m*, of the sample analyzed was calculated using Eq. (1).

1 $$m = \frac{{m}_{1}{m}_{3}}{{m}_{2}}$$

where *m*_*1*_ is the mass of the initial sample subject to heating or concentration, *m*_*2*_ is the mass of the heated or concentrated sample, and *m*_*3*_ is the mass of heated or concentrated sample transferred in the vial. The radiation recoveries of the heating procedures, *η*, were determined by measurement of spiked ^241^Am and ^90^Sr/^90^Y samples.

A direct counting method was generally adopted for the measurement of radioactive wastewater. After pH adjustment, we transferred 5 mL of the aliquot into the vial, closed it, and shook it vigorously to remove most of the dissolved ^222^Rn. Then the sample was mixed with 15 mL of cocktail for measurement. In addition, 50–100 mL of water sample was completely dried and weighed to obtain the solid residue quality of the sample.

### Calculations

Alpha/beta activity concentrations, standard uncertainties, and detection limits are calculated according to the equations shown in ASTM D7283-17^7^ as follows.

2 $${\varepsilon }_{\alpha \alpha } = \frac{{R}_{\alpha \alpha }-{R}_{\alpha b}}{{C}_{\alpha } {V}_{s\alpha }}, \quad \varepsilon_{\alpha \beta }= \frac{{R}_{\alpha \beta }-{R}_{\beta b}}{{C}_{\alpha } {V}_{s\alpha }},\quad \varepsilon_{\beta \beta }= \frac{{R}_{\beta \beta }-{R}_{\beta b}}{{C}_{\beta } {V}_{s\beta }},\quad \varepsilon_{\beta \alpha }= \frac{{R}_{\beta \alpha }-{R}_{\alpha b}}{{C}_{\beta } {V}_{s\beta }}$$

where *ε*_*αα*_ is detection efficiency of the ^241^Am standard aliquot in the regions of interest (ROI) for alpha, *ε*_*αβ*_ is detection efficiency of the ^241^Am standard aliquot in the beta ROI, *ε*_*ββ*_ is detection efficiency of the ^90^Sr/^90^Y standard aliquot in the beta ROI, *ε*_*βα*_ is detection efficiency of the ^90^Sr/^90^Y standard aliquot in the alpha ROI. *R*_*αα*_ is count rate of the ^241^Am standard aliquot in the alpha ROI (s^−1^), *R*_*αβ*_ is count rate of the ^241^Am standard aliquot in the beta ROI (s^−1^), *R*_*ββ*_ is count rate of the ^90^Sr/^90^Y standard aliquot in the beta ROI (s^−1^), *R*_*βα*_ is count rate of the ^90^Sr/^90^Y standard aliquot in the alpha ROI (s^−1^), *R*_*αb*_ is count rate of the background sample in the alpha ROI (s^−1^), *R*_*βb*_ is count rate of the background sample in the beta ROI (s^−1^). *C*_*α*_ is activity concentration of the ^241^Am standard solution (Bq g^−1^), *C*_*β*_ is activity concentration of the ^90^Sr/^90^Y standard solution (Bq g^−1^), *V*_*sα*_ is the volume of the ^241^Am standard solution added to the vial (g), *V*_*sβ*_ is the volume of the ^90^Sr/^90^Y standard solution added to the vial (g).

3 $${X}_{\alpha }=\frac{{\varepsilon }_{\alpha \beta }}{{\varepsilon }_{\alpha \alpha }},\quad {X}_{\beta }=\frac{{\varepsilon }_{\beta \alpha }}{{\varepsilon }_{\beta \beta }}$$

where *X*_*α*_ is alpha-to-beta spillover factor and *X*_*β*_ is beta-to-alpha spillover factor.

The net count rates in the alpha ROI and beta ROI are calculated as follows.

4 $$R_{\alpha } \, = \,R_{\alpha \alpha } - R_{\alpha b} ,\quad R_{\beta } \, = \,R_{\beta \beta } - R_{\beta b}$$

5 $${R}_{\alpha }^{\mathrm{^{\prime}}}=\frac{{R}_{\alpha }-{R}_{\beta }{X}_{\beta }}{1-{X}_{\alpha }{X}_{\beta }},\quad {R}_{\beta }^{\mathrm{^{\prime}}}=\frac{{R}_{\beta }-{R}_{\alpha }{X}_{\alpha }}{1-{X}_{\alpha }{X}_{\beta }}$$

where *R*_*α*_ is net count rate of the sample aliquot in the alpha ROI (s^−1^) and *R*_*β*_ is net count rate of the sample aliquot in the beta ROI (s^−1^). ${R}_{\alpha }^{^{\prime}}$ is alpha count rate corrected for spillover (s^−1^) and ${R}_{\beta }^{^{\prime}}$ is beta count rate corrected for spillover (s^−1^).

The sample gross alpha/beta activity concentrations are calculated from the following:

6 $$AC\alpha =\frac{{R}_{\alpha }^{\mathrm{^{\prime}}}}{{\varepsilon }_{\alpha \alpha } V},\quad AC\beta = \frac{{R}_{\beta }^{\mathrm{^{\prime}}}}{{\varepsilon }_{\beta \beta } V}$$

where *AC*_*α*_ is sample gross alpha activity concentration (Bq L^−1^) and *AC*_*β*_ is sample gross beta activity concentration (Bq L^−1^). *V* is sample aliquot volume (L).

The standard uncertainties of these parameters and alpha/beta activity concentrations are calculated using equations as specified in ISO/IEC Guide 98–3: 2008^29^ as follows.

$$u\left(R_\alpha \right)=\sqrt{\frac{{R}_{\alpha \alpha }}{{t}_{s}}+ \frac{{R}_{\alpha b}}{{t}_{b}}},\quad u\left(R_{\alpha \beta }-R_{\beta b}\right)=\sqrt{\frac{{R}_{\alpha \beta }}{{t}_{s}}+ \frac{{R}_{\beta b}}{{t}_{b}}},$$

7 $$u\left(R_\beta \right)=\sqrt{\frac{{R}_{\beta \beta }}{{t}_{s}}+ \frac{{R}_{\beta b}}{{t}_{b}}},\quad u(R_{\beta \alpha }-R_{\alpha b}) = \sqrt{\frac{{R}_{\beta \alpha }}{{t}_{s}}+ \frac{{R}_{\alpha b}}{{t}_{b}}}$$

$$u\left(\varepsilon_{\alpha \alpha }\right)\hspace{0.17em}=\hspace{0.17em}\varepsilon_{\alpha \alpha }\sqrt{\frac{{u}^{2}\left({R}_{\alpha }\right)}{{\left({R}_{\alpha \alpha }-{R}_{\alpha b}\right)}^{2}}+ \frac{{u}^{2}\left({C}_{\alpha }\right)}{{C}_{\alpha }^{2}}+ \frac{{u}^{2}\left({V}_{s\alpha }\right)}{{V}_{s\alpha }^{2}}},\quad u(\varepsilon_{ \alpha \beta })=\hspace{0.17em}\varepsilon_{ \alpha \beta} \sqrt{\frac{{u}^{2}\left({R}_{\alpha \beta }-{R}_{\beta b}\right)}{{\left({R}_{\alpha \beta }-{R}_{\beta b}\right)}^{2}}+ \frac{{u}^{2}\left({C}_{\alpha }\right)}{{C}_{\alpha }^{2}}+ \frac{{u}^{2}\left({V}_{s\alpha }\right)}{{V}_{s\alpha }^{2}},}$$

8 $$\mathrm{u}\left(\mathrm{\varepsilon_{ \beta \beta }}\right)\hspace{0.17em}=\hspace{0.17em}\mathrm{\varepsilon_{ \beta \beta} }\sqrt{\frac{{u}^{2}\left({R}_{\beta }\right)}{{\left({R}_{\beta \beta }-{R}_{\beta b}\right)}^{2}}+ \frac{{u}^{2}\left({C}_{\beta }\right)}{{C}_{\beta }^{2}}+ \frac{{u}^{2}\left({V}_{s\beta }\right)}{{V}_{s\beta }^{2}}},\quad \mathrm{u}(\mathrm{\varepsilon_{ \beta \alpha }})\hspace{0.17em}=\hspace{0.17em}\mathrm{\varepsilon_{ \beta \alpha } }\sqrt{\frac{{u}^{2}\left({R}_{\beta \alpha }-{R}_{\alpha b}\right)}{{\left({R}_{\beta \alpha }-{R}_{\alpha b}\right)}^{2}}+ \frac{{u}^{2}\left({C}_{\beta }\right)}{{C}_{\beta }^{2}}+ \frac{{u}^{2}\left({V}_{s\beta }\right)}{{V}_{s\beta }^{2}}}$$

9 $$u\left(X_\alpha \right)\hspace{0.17em}=\hspace{0.17em}X_\alpha \sqrt{\frac{{u}^{2}\left({\varepsilon }_{\alpha \beta }\right)}{{\varepsilon }_{\alpha \beta }^{2}}+ \frac{{u}^{2}\left({\varepsilon }_{\alpha \alpha }\right)}{{\varepsilon }_{\alpha \alpha }^{2}}},\quad u(X_\beta )\hspace{0.17em}=\hspace{0.17em}X_\beta \sqrt{\frac{{u}^{2}({\varepsilon }_{\beta \alpha })}{{\varepsilon }_{\beta \alpha }^{2}}+ \frac{{u}^{2}({\varepsilon }_{\beta \beta })}{{\varepsilon }_{\beta \beta }^{2}}}$$

$$u_c\left({R}_{\alpha }^{\mathrm{^{\prime}}}\right)=\sqrt{\frac{{u}^{2}\left({R}_{\alpha }\right)+ {X}_{\beta }^{2}{u}^{2}\left({R}_{\beta }\right)+ {R}_{\alpha }^{\mathrm{^{\prime}}2}{X}_{\beta }^{2}{u}^{2}\left({X}_{\alpha }\right)+ {R}_{\beta }^{\mathrm{^{\prime}}2}{u}^{2}\left({X}_{\beta }\right)}{1-{X}_{\alpha }{X}_{\beta }}},$$

10 $$u_c({R}_{\beta }^{\mathrm{^{\prime}}})=\sqrt{\frac{{u}^{2}\left({R}_{\beta }\right)+ {X}_{\alpha }^{2}{u}^{2}\left({R}_{\alpha }\right)+ {R}_{\beta }^{\mathrm{^{\prime}}2}{X}_{\alpha }^{2}{u}^{2}\left({X}_{\beta }\right)+ {R}_{\alpha }^{\mathrm{^{\prime}}2}{u}^{2}({X}_{\alpha })}{1-{X}_{\alpha }{X}_{\beta }}}$$

$$u_c\left(AC_\alpha \right)=\sqrt{\frac{{u}_{c}^{2}\left({R}_{\alpha }^{\mathrm{^{\prime}}}\right)}{{\varepsilon }_{\alpha \alpha }^{2}\cdot {V}^{2}}+ {AC}_{\alpha }^{2}\cdot \left(\frac{{u}^{2}\left(V\right)}{{V}^{2}} + \frac{1+ {X}_{\alpha }{X}_{\beta }}{1-{X}_{\alpha }{X}_{\beta }}\cdot \frac{{u}^{2}\left({\varepsilon }_{\alpha \alpha }\right)}{{\varepsilon }_{\alpha \alpha }^{2}} \right)},$$

11 $$u_c(AC_\beta ) = \sqrt{\frac{{u}_{c}^{2}({R}_{\beta }^{\mathrm{^{\prime}}})}{{\varepsilon }_{\beta \beta }^{2}\cdot {V}^{2}}+ {AC}_{\beta }^{2}\cdot \left(\frac{{u}^{2}\left(V\right)}{{V}^{2}} + \frac{1+ {X}_{\alpha }{X}_{\beta }}{1-{X}_{\alpha }{X}_{\beta }}\cdot \frac{{u}^{2}({\varepsilon }_{\beta \beta })}{{\varepsilon }_{\beta \beta }^{2}} \right)}$$

where *u(R*_*α*_*)* is standard uncertainty of the net count rate of the sample aliquot in the alpha ROI, *u(R*_*β*_*)* is standard uncertainty of the net count rate of the sample aliquot in the beta ROI, *u(R*_*αβ*_*—R*_*βb*_*)* is standard uncertainty of the net count rate of the ^241^Am standard aliquot in the beta ROI, and *u(R*_*βα*_*—R*_*αb*_*)* is standard uncertainty of the net count rate of the ^90^Sr/^90^Y standard aliquot in the alpha ROI. *u(ε*_*αα*_*)* is standard uncertainty of the alpha particle detection efficiency in the alpha ROI, *u(ε*_*αβ*_*)* is standard uncertainty of the alpha particle detection efficiency in the beta ROI, *u(ε*_*ββ*_*)* is standard uncertainty of the beta particle detection efficiency in the beta ROI, and *u(ε*_*βα*_*)* is standard uncertainty of the beta particle detection efficiency in the alpha ROI. *u(X*_*α*_*)* is standard uncertainty of the alpha spillover factor, and *u(X*_*β*_*)* is standard uncertainty of the beta spillover factor. *u*_*c*_*(*${R}_{\alpha }^{^{\prime}}$*)* is the combined standard uncertainty of the alpha count rate corrected for spillover, and *u*_*c*_*(*${R}_{\beta }^{^{\prime}}$*)* is the combined standard uncertainty of the beta count rate corrected for spillover. *u*_*c*_*(AC*_*α*_*)* is the combined standard uncertainty of the sample gross alpha activity concentration, and *u*_*c*_*(AC*_*β*_*)* is the combined standard uncertainty of the sample gross beta activity concentration.

The expanded uncertainties of the parameters and alpha/beta activity concentrations are calculated using equations as follows.

12 $$U\, = \,k \, \cdot \, u\left( i \right),\quad U\, = \,k \, \cdot \, u_{c} \left( i \right)$$

where *U* is expanded uncertainty, *k* is coverage factor, *u(i)* and *u*_*c*_*(i)* are standard uncertainty and combined standard uncertainty of the measured values referred above, respectively. In our work, the value of *k* takes 1.

Minimum detectable concentrations (MDC) for gross alpha and gross beta activity concentrations are calculated using equations as follows.

13 $$\mathrm{MDC_\alpha }= \frac{2.71\cdot \frac{(1+{\mathrm{X}}_{\mathrm{\alpha }}{\mathrm{X}}_{\upbeta }^{2})}{{\mathrm{t}}_{\mathrm{s }}\cdot (1-{\mathrm{X}}_{\mathrm{\alpha }}{\mathrm{X}}_{\upbeta })}+3.29 \cdot \sqrt{\frac{{\mathrm{AC}}_{\upbeta } \cdot \mathrm{ V }\cdot {\upvarepsilon }_{\mathrm{\beta \alpha }} \cdot (1+{\mathrm{X}}_{\upbeta })}{{\mathrm{t}}_{\mathrm{s}}}+ \left({\mathrm{R}}_{\mathrm{\alpha b}}+ {\mathrm{X}}_{\upbeta }^{2}{\mathrm{R}}_{\mathrm{\beta b}}\right) \cdot (\frac{1}{{\mathrm{t}}_{\mathrm{s}}}+ \frac{1}{{\mathrm{t}}_{\mathrm{b}}} )}}{{\upvarepsilon }_{\mathrm{\alpha \alpha }} \cdot \mathrm{V }\cdot (1-{\mathrm{X}}_{\mathrm{\alpha }}{\mathrm{X}}_{\upbeta })}$$

14 $$\mathrm{MDC_\beta }= \frac{2.71\cdot \frac{(1+{\mathrm{X}}_{\upbeta }{\mathrm{X}}_{\mathrm{\alpha }}^{2})}{{\mathrm{t}}_{\mathrm{s }}\cdot (1-{\mathrm{X}}_{\mathrm{\alpha }}{\mathrm{X}}_{\upbeta })}+3.29 \cdot \sqrt{\frac{{\mathrm{AC}}_{\mathrm{\alpha }} \cdot \mathrm{ V }\cdot {\upvarepsilon }_{\mathrm{\alpha \beta }} \cdot (1+{\mathrm{X}}_{\mathrm{\alpha }})}{{\mathrm{t}}_{\mathrm{s}}}+ \left({\mathrm{R}}_{\mathrm{\beta b}}+ {\mathrm{X}}_{\mathrm{\alpha }}^{2}{\mathrm{R}}_{\mathrm{\alpha b}}\right) \cdot (\frac{1}{{\mathrm{t}}_{\mathrm{s}}}+ \frac{1}{{\mathrm{t}}_{\mathrm{b}}} )}}{{\upvarepsilon }_{\mathrm{\beta \beta }} \cdot \mathrm{V }\cdot (1-{\mathrm{X}}_{\mathrm{\alpha }}{\mathrm{X}}_{\upbeta })}$$

where t_s_ is sample aliquot count time in seconds, and t_b_ is background aliquot count time in seconds.

### Determination of the γ-radionuclides by gamma spectrometry

Two standard aqueous solutions were used for energy and efficiency calibrations of high pure germanium gamma spectrometry^30^. One solution contained ^241^Am, ^133^Ba, ^137^Cs, ^60^Co, and the other contained ^40^K in two sealed cylindrical plastic containers (7.5 cm in diameter and 7 cm in height). They were all certified by the national first-class ionizing radiation metrology station with the values of (3.55 ± 0.17) × 10^3^ Bq, (1.15 ± 0.05) × 10^3^ Bq, (3.18 ± 0.11) × 10^3^ Bq, (1.31 ± 0.06) × 10^3^ Bq, and (1.19 ± 0.05) × 10^3^ Bq (with coverage factor *k* = 2 for 95% confidence) for ^241^Am, ^133^Ba, ^137^Cs, ^60^Co and ^40^K, respectively. The full-energy peak efficiency *ε*_*f*_ is calculated as follows:

15 $$\varepsilon_ f = \frac{{R}_{net}}{A \times I}$$

where *R*_*net*_ is net gamma-ray count rate in the full-energy peak of interest (s^−1^), *A* is activity of the standard source (Bq) and *I* is absolute gamma intensity for the specific gamma-ray emission.

For samples with gross alpha or gross beta activity concentrations exceeding the management limits, gamma spectrometry was used to determine radionuclides such as U, Th, Ra, ^137^Cs, ^40^K, etc. Samples of about 240 g were transferred to the specimen containers and measured in the same manner as were done during calibration. The counting time of the sample was 1440 min. The gamma nuclide activity concentration in the sample *AC*_*γ*_ (Bq) is calculated as follows:

16 $$AC_\gamma = \frac{{R}_{net}}{\varepsilon \times V \times I}$$

where *V* is test specimen volume (L). Because the radionuclides of interest have medium or long half-lives, their decay corrections are not taken into account in the calculations.

The combined standard uncertainty of the nuclide activity concentration *u*_*c*_*(AC*_*γ*_*)* and minimum detectable concentrations (MDC_γ_) are calculated using equations as follows.

17 $$u_c(AC_\gamma ) = \sqrt{\frac{{u}^{2}({R}_{net})}{{\varepsilon }^{2} \times {V}^{2} \times {I}^{2}}+ {AC}_{\gamma }^{2} \times \left(\frac{{u}^{2}(\varepsilon )}{{\varepsilon }^{2}}+ \frac{{u}^{2}(V)}{{V}^{2}}+ \frac{{u}^{2}(I)}{{I}^{2}}\right)}$$

18 $$\mathrm{MDC_\gamma }= \frac{\frac{2.71}{{t}_{s}} +3.29 \sqrt{\frac{{R}_{b}}{{t}_{s}} \times \left( \frac{{n}_{p}}{{n}_{b}} + \frac{{n}_{p}^{2}}{{n}_{b}^{2}} \right)} }{\varepsilon \times V \times I}$$

where *u(R*_*net*_*)* is the standard uncertainty of the net counting rate, *u(ε)* is the standard uncertainty of the detector efficiency, *u(V)* is the standard uncertainty of the sample volume measurement, *u(I)* is the standard uncertainty of the absolute gamma intensity. *t*_*s*_ is the counting time of sample (s), *R*_*b*_ is baseline background count rate (s^−1^), *n*_*p*_ is number of channels in the photopeak, and *n*_*b*_ is number of channels used in the baseline subtraction.

If necessary, more specific analytical strategies would be developed.

### Sample collection

In this paper, five types of actual water samples were collected to test the LSC method, which were drinking water, groundwater, geothermal water, seawater, and wastewater. Among them, drinking water, groundwater and seawater were collected from Ningde City, Fujian Province, China. Geothermal water was collected from a hot spring center in Beijing, China. And wastewater was collected from the wastewater storage tank of a nuclear facility in China. The water sample was put in a 10 L polyethylene plastic container and transported back to the laboratory for pretreatment within 5 days. In the laboratory, the water sample was firstly filtered through a membrane with φ 0.45 μm to remove suspended particles. Then, it was treated according to the procedure described in the section of Sample pretreatment for LSC measurement.

## Results and discussion

### Establishment of LSC method

#### Optimization of counting conditions

Firstly, the optimal counting regions were determined. The representative alpha nuclides (^241^Am and ^239^Pu) and beta nuclides (^3^H, ^14^C, ^63^Ni, ^40^K, and ^90^Sr/^90^Y) were selected for measurements, and their LSC spectrums were shown in Figure S1. In addition to the spectral locations of nuclides, the figure of merit was also considered for counting region optimization. The figure of merit is defined as the square of the percent counting efficiency of the radionuclide of interest divided by the background count rate expressed in counts per minute, which is an important parameter used to optimize LSC performance, particularly for low activity samples^21^. According to LSC spectrums and the figure of merit (E^2^ B^−1^), the ROIs for alpha and beta counting were from 400–800 channel and 250–950 channel, respectively. All alpha events with the energy of 4–8 MeV and all beta events except tritium can be detected in the ranges.

**Figure 1:**
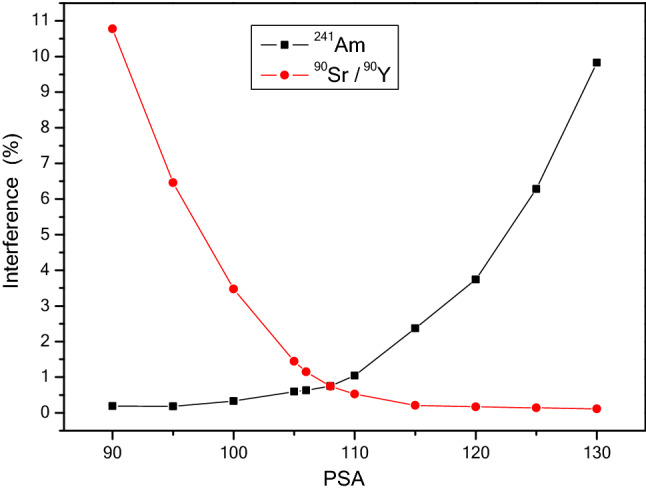
Calibration curves of alpha and beta interferences under different PSA settings.

Then the alpha and beta spillover factors for each PSA setting were calculated, and the variation curves along with the PSA were shown in Fig. 1. From Fig. 1, the optimal PSA value is 108, and the minimum interference value is 1.51%. Other optimal parameters, including efficiencies of ^241^Am and ^90^Sr/^90^Y standard aliquot and background sample count rates, were all shown in Table 2.

**Table 2:** Optimal counting parameters obtained for alpha and beta standard aliquots used in the calibration.

Parameters	Value ± U *
*ε*_*αα*_ (%)	100.55 ± 0.65
*ε*_*αβ*_ (%)	0.76 ± 0.04
*ε*_*ββ*_ (%)	94.90 ± 0.49
*ε*_*βα*_ (%)	0.71 ± 0.05
*R*_*αb*_ (min^−1^)	0.377 ± 0.019
*R*_*βb*_ (min^−1^)	3.146 ± 0.056
*X*_*α*_ (%)	0.758 ± 0.041
*X*_*β*_ (%)	0.752 ± 0.048
SQP(E)	803.8 ± 5.3

*U is the expanded uncertainty corresponding to k = 1.

#### Optimization of pH range

**Figure 2:**
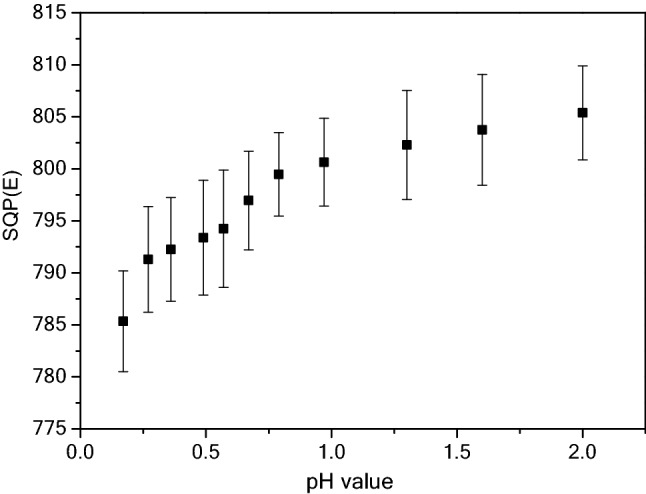
Variation curve of SQP(E) value at different pH values in HNO_3_ medium.

After sampling, a certain amount of acid needs to be added to the water sample for minimization the loss of radioactivity. Generally, nitric acid is chosen for acidification treatment in the practical application in radioactivity monitoring. However, the addition of nitric acid increases the quenching for the LSC measurement. As shown in Fig. 2, the change tendency of sample quenching at different pH values was investigated. SQP(E) values show a gradual downward tendency with the decrease of pH values. As a laboratory controllable factor, acidification should not only minimize the adsorption of radioactive materials, but also minimize the influence of quenching. From Fig. 2, the optimal pH range is 1.6–2 for the LSC measurement. In this range, the SQP(E) values are all greater than 803, indicating that the quenching effect of the sample is still small.

#### Calibration of the saline water sample

**Figure 3:**
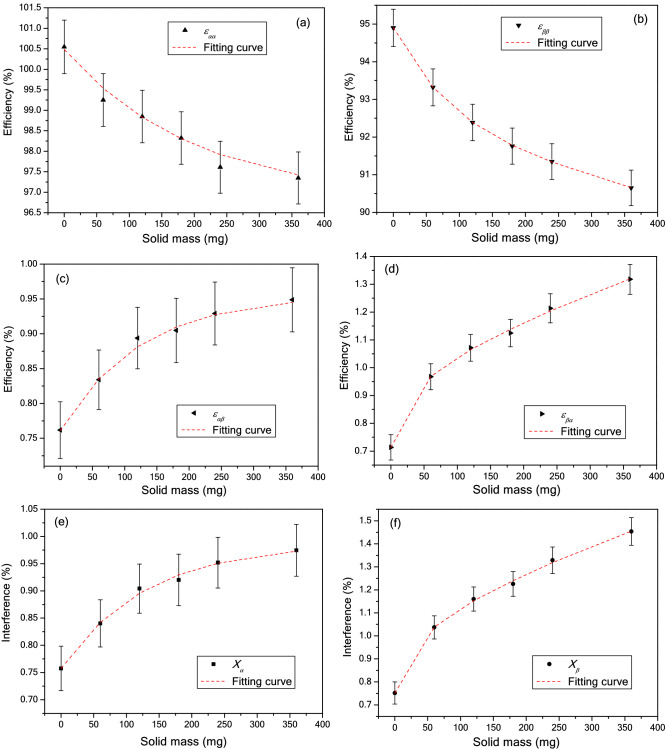
Calibration curves of *ε*_*aa*_ (**a**), *ε*_*ββ*_ (**b**), *ε*_*αβ*_ (**c**), *ε*_*βα*_ (**d**), *X*_*α*_ (**e**) and *X*_*β*_ (**f**) vs. solid mass under the condition of PSA = 108.

Solid salts dissolved in water samples cause a certain degree of quenching, which will affect the detection efficiencies and spillover factors of alpha and beta. Therefore, we investigated the effect of different salt content on alpha–beta separation. 1 g ^241^Am or ^90^Sr/^90^Y standard solution was mixed with varying volumes of SS solution and measured under different PSA settings. The results show that the SQP(E) value change was not sensitive to increasing salt content. When salt content increased from 60 to 360 mg, SQP(E) value decreased slowly from 797.7 ± 4.9 to 791.4 ± 5.2, and the optimal PSA values of these samples fluctuated in a narrow range from 106 to 109, but the sum of *X*_*α*_ and *X*_*β*_ increased. The graphs of *ε*_*aa*_, *ε*_*αβ*_, *ε*_*ββ*_, *ε*_*βα*_, *X*_*α*_, and *X*_*β*_ concerning salt mass are shown in Fig. 3. With the increase of solid mass, the detection efficiencies of ^241^Am and ^90^Sr/^90^Y decreased from 100.5% to 97.3% and 94.9% to 90.6%, respectively, while the misclassification efficiencies and spillover factors of α/β both increased. The fitting curves of these six parameters with the change of solid mass are listed in Table 3.

**Table 3:** Fitting curves of *ε*_*aa*_, *ε*_*ββ*_, *ε*_*αβ*_, *ε*_*βα*_, *X*_*α*_ and *X*_*β*_* vs*. solid mass (*m*_*r*_, mg).

Fitting curves	*R*^*2*^
*ε*_*aa*_ = 96.814 + 3.6717 exp ( – 0.005 *m*_*r*_ )	0.971
*ε*_*ββ*_ = 2.6155 exp ( – *m*_*r*_ / 86.9253 ) + 62,068.849 exp ( – *m*_*r*_ / 1.3341 × 10^7^ ) – 61,976.56	0.998
*ε*_*αβ*_ = 0.956 – 0.195 exp ( – 0.008 *m*_*r*_ )	0.988
*ε*_*βα*_ = – 0.1908 exp ( – *m*_*r*_ / 26.9048 ) – 1.0292 exp ( – *m*_*r*_ / 697.1461 ) + 1.9335	0.993
*X*_*α*_ = 0.9877 – 0.23 exp ( – 0.00763 *m*_*r*_ )	0.991
*X*_*β*_ = – 1.2121 exp ( – *m*_*r*_ / 687.216 ) – 0.2093 exp ( – *m*_*r*_ / 27.9307 ) + 2.1732	0.994

### Validation for LSC method

#### The spiked samples

The ^241^Am, ^90^Sr-^90^Y, and ^40^K standard solutions were mixed with nitric acid carrier or SS solution to prepare three series of spiked samples with different activity levels for method validation. The samples are numbered according to the activity levels. If it is greater than 5 Bq, indicated by H, being between 0.5 Bq to 5 Bq will be indicated by M, and being less than 0.5 Bq, indicated by L. The measurement results are shown in Tables 4, 5, 6, and the comparison graphs of the activity relative deviations of alpha and beta for three series of spiked samples are shown in Figure S2. The pure spiked sample was prepared by ^241^Am, ^90^Sr-^90^Y, or ^40^K standard solution with nitric acid carrier solution, and the mixed spiked samples were prepared by mixing ^241^Am and ^90^Sr-^90^Y standard solutions. For the pure spiked samples (Table 4), the activity deviations were within 7%. For the mixed spiked samples, the activity ratio of gross alpha and gross beta ranged from 1:102 to 67:1. And the relative deviations of gross alpha and gross beta were less than 8% and less than 17%, respectively. For the case of non-quenching samples (Table 5), when the activity ratio of gross alpha and gross beta was about 1, such as L_α_L_β_, M_α_M_β_, and H_α_H_β_ samples, the deviations of gross alpha and gross beta fluctuated little. When the activity ratio of gross alpha and gross beta increased from 1:6 to 1:85, such as M_α_H_β_, L_α_M_β_, and L_α_H_β_ samples, the alpha deviations was increased from 0.6 to 7.6% due to the effect of high beta activity. For the samples with high alpha and low beta activities, the gross beta activity was less affected by gross alpha activity, and the deviation was less than 5%. Bhade SPD and Zapata Garcia D have also reported similar phenomena^11,12^.

**Table 4:** Validation results of pure spiked samples of ^40^K, ^241^Am, and ^90^Sr/^90^Y standard solution.

Sample No	Solid mass (mg)	Theoretical alpha activity (Bq)^a^	Measured alpha activity (Bq)^b^	Alpha activity deviation (%)	Theoretical beta activity (Bq)^a^	Measured beta activity (Bq)^b^	Beta activity deviation (%)
M(^241^Am)	0	2.05 ± 0.01	1.97 ± 0.02	− 3.9	–	≤ MDA = 7.62 E−03^c^	–
M(^90^Sr)	0	–	≤ MDA = 4.41 E−03^c^	–	3.12 ± 0.01	3.10 ± 0.08	− 0.6
M(^40^K)	139	–	≤ MDA = 4.60 E−03^c^	–	2.11 ± 0.05	2.17 ± 0.06	2.8
L(^241^Am)	0	(2.08 ± 0.01) E−01	(2.22 ± 0.05) E−01	6.7	–	≤ MDA = 6.99 E−03^c^	–
L(^90^Sr)	0	–	≤ MDA = 2.66 E−03^c^	–	(3.22 ± 0.01) E−01	(3.35 ± 0.76) E−01	4.0
L(^40^K)	14	–	≤ MDA = 2.58 E−03^c^	–	(2.03 ± 0.05) E−01	(2.11 ± 0.67) E−01	3.9

^a^Data obtained by multiplying the activity of the standard solution and the mass added. The expanded uncertainty corresponds to k = 1.

^b^The expanded uncertainty corresponds to k = 1.

^c^MDA (minimum detectable activity, Bq) = MDC (minimum detectable concentration, Bq/L) × *V* (sample aliquot volume, L).

**Table 5:** Validation results of mixed spiked samples of ^241^Am, and ^90^Sr/^90^Y standard solution (solid mass = 0).

Sample No	α/β activity ratio	Theoretical alpha activity (Bq)^a^	Measured alpha activity (Bq)^b^	Alpha activity deviation (%)	Theoretical beta activity (Bq)^a^	Measured beta activity (Bq)^b^	Beta activity deviation (%)
L_α_L_β_	1: 1.5	(2.30 ± 0.01) E−01	(2.26 ± 0.05) E−01	− 1.7	(3.53 ± 0.01) E−01	(3.55 ± 0.06) E−01	0.6
L_α_L_β_	1: 1.9	(6.36 ± 0.05) E−02	(6.44 ± 0.28) E−02	1.3	(1.22 ± 0.01) E−01	(1.27 ± 0.04) E−01	4.1
H_α_H_β_	1: 1.5	8.26 ± 0.03	8.15 ± 0.04	− 1.3	(1.25 ± 0.01) E + 01	(1.24 ± 0.02) E + 01	− 0.8
L_α_M_β_	1: 12.8	(2.45 ± 0.01) E−01	(2.49 ± 0.05) E−01	1.6	3.13 ± 0.01	3.12 ± 0.05	− 0.3
L_α_H_β_	1: 50.2	(2.51 ± 0.01) E−01	(2.62 ± 0.06) E−01	4.4	(1.26 ± 0.01) E + 01	(1.24 ± 0.02) E + 01	− 1.6
L_α_H_β_	1: 84.5	(1.48 ± 0.01) E−01	(1.59 ± 0.05) E−01	7.4	(1.25 ± 0.01) E + 01	(1.23 ± 0.02) E + 01	− 1.4
M_α_H_β_	1: 5.9	2.12 ± 0.01	2.13 ± 0.02	0.5	(1.25 ± 0.01) E + 01	(1.23 ± 0.02) E + 01	− 1.6
M_α_M_β_	1: 1.5	2.10 ± 0.01	2.02 ± 0.02	− 3.8	3.15 ± 0.01	3.06 ± 0.02	− 2.9
M_α_L_β_	6.0: 1	2.11 ± 0.01	2.02 ± 0.02	− 4.3	(3.49 ± 0.01) E−01	(3.60 ± 0.03) E−01	3.2
M_α_L_β_	24.1: 1	4.10 ± 0.02	4.08 ± 0.03	− 0.5	(1.70 ± 0.01) E−01	(1.76 ± 0.02) E−01	3.5
H_α_L_β_	22.6: 1	8.30 ± 0.03	8.21 ± 0.04	− 1.1	(3.67 ± 0.01) E−01	(3.65 ± 0.03) E−01	− 0.5
H_α_L_β_	66.1: 1	9.92 ± 0.04	(1.02 ± 0.02) E + 01	2.8	(1.50 ± 0.01) E−01	(1.43 ± 0.02) E−01	− 4.7
H_α_M_β_	2.6: 1	8.21 ± 0.03	8.08 ± 0.04	− 1.6	3.14 ± 0.01	3.06 ± 0.02	− 2.5

^a^Data obtained by multiplying the activity of the standard solution and the mass added. The expanded uncertainty corresponds to k = 1.

^b^The expanded uncertainty corresponds to k = 1.

**Table 6:** Validation results of mixed spiked samples of ^241^Am, and ^90^Sr/^90^Y standard solution in simulated saline water.

Sample No	Solid mass (mg)	α/β activity ratio	Theoretical alpha activity (Bq)^a^	Measured alpha activity (Bq)^b^	Alpha activity deviation (%)	Theoretical beta activity (Bq)^a^	Measured beta activity (Bq)^b^	Beta activity deviation (%)
L_α_L_β_	180	1: 1.6	(1.09 ± 0.01) E−01	(1.04 ± 0.04) E−01	− 4.6	(1.71 ± 0.01) E−01	(1.46 ± 0.04) E−01	− 14.6
L_α_L_β_	360	1: 1.9	(9.23 ± 0.05) E−02	(8.90 ± 0.33) E−02	− 3.6	(1.77 ± 0.01) E−01	(1.53 ± 0.04) E−01	− 13.6
H_α_H_β_	180	1: 1.5	8.23 ± 0.03	8.28 ± 0.04	0.6	(1.25 ± 0.01) E + 01	(1.28 ± 0.02) E + 01	2.4
H_α_H_β_	360	1: 1.5	8.21 ± 0.03	8.34 ± 0.04	1.6	(1.26 ± 0.01) E + 01	(1.29 ± 0.02) E + 01	2.4
L_α_M_β_	180	1: 16.7	(2.06 ± 0.01) E−01	(1.99 ± 0.05) E−01	− 3.4	3.43 ± 0.01	3.48 ± 0.06	1.5
L_α_M_β_	360	1: 16.0	(2.26 ± 0.01) E−01	(2.16 ± 0.05) E−01	− 4.4	3.61 ± 0.01	3.43 ± 0.05	− 5.0
L_α_H_β_	180	1: 87.5	(1.44 ± 0.01) E−01	(1.37 ± 0.05) E−01	− 4.9	(1.26 ± 0.01) E + 01	(1.28 ± 0.02) E + 01	1.7
L_α_H_β_	360	1: 101.6	(1.24 ± 0.01) E−01	(1.17 ± 0.06) E−01	− 5.6	(1.26 ± 0.01) E + 01	(1.28 ± 0.02) E + 01	1.6
M_α_M_β_	180	1.3: 1	4.11 ± 0.02	4.14 ± 0.03	0.7	3.21 ± 0.01	3.19 ± 0.02	− 0.6
M_α_M_β_	360	1.3: 1	4.13 ± 0.02	4.17 ± 0.03	1.0	3.17 ± 0.01	3.26 ± 0.02	2.8
M_α_L_β_	180	24.7: 1	4.10 ± 0.02	4.17 ± 0.03	1.8	(1.66 ± 0.01) E−01	(1.44 ± 0.02) E−01	− 13.3
M_α_L_β_	360	23.0: 1	4.10 ± 0.02	4.21 ± 0.03	2.7	(1.78 ± 0.01) E−01	(1.58 ± 0.02) E−01	− 11.3
H_α_L_β_	180	67.3: 1	(1.05 ± 0.01) E + 01	(1.04 ± 0.02) E + 01	− 1.0	(1.56 ± 0.01) E−01	(1.30 ± 0.03) E−01	− 16.7
H_α_L_β_	360	64.3: 1	(1.01 ± 0.01) E + 01	(1.05 ± 0.02) E + 01	4.0	(1.57 ± 0.01) E−01	(1.36 ± 0.03) E−01	− 13.4
H_α_M_β_	180	2.6: 1	8.21 ± 0.03	8.22 ± 0.04	0.2	3.12 ± 0.01	3.07 ± 0.02	− 1.6
H_α_M_β_	360	2.8: 1	8.23 ± 0.03	8.28 ± 0.04	0.6	2.99 ± 0.01	3.06 ± 0.02	2.3

^a^Data obtained by multiplying the activity of the standard solution and the mass added. The expanded uncertainty corresponds to k = 1. ^b^The expanded uncertainty corresponds to k = 1.

When soluble salts exist in solutions (Table 6), for medium and high activity samples, such as M_α_M_β_ and H_α_H_β_, the activity deviations of gross alpha and gross beta changed little. But for low activity samples, such as L_α_L_β_, the gross alpha and gross beta activities also showed negative deviations. The gross alpha activities for the samples of L_α_M_β_ and L_α_H_β_ and the gross beta activities for the samples of M_α_L_β_ and H_α_L_β_ showed negative variation in varying degrees, which indicated that the calibration curve had some variation for these samples. It is known that the optimal PSA value is affected by many factors, one of which is the activity concentration of standard nuclide. When the activity or activity ratio of standard solutions used for calibration is greatly different from the state of the measured sample, the optimal PSA value will occur to displacement^11,16^. It also makes the values of calibration parameters such as *X*_*α*_ and *X*_*β*_ deviate. The larger the quenching of the sample accompanied with the more significant the displacement of PSA value, which will cause the more considerable the deviation of the calculated results occurred. We tried to avoid quenching in the analysis of samples, and monitor the quenching level by SQP(E) value so that the activity deviations of samples can be controlled within 30%, which satisfies the quality control requirements for routine monitoring.

#### The intercomparison samples

Our laboratory participated in proficiency tests for the gross alpha/beta of water samples organized by the National Institute for Radiological Protection, China CDC, in 2019 and 2020. The water samples were collected from the groundwater in the areas with relatively high levels of natural radioactivity. After that, the samples were transported to the laboratory for filtration, nitric acid addition, stirring, packing and delivery. The total numbers of institutions participating in the proficiency test were 121 and 127, respectively. The results were evaluated by the Z-Score method, and the reference value was measured by the designated organizations.

The samples we received were colorless, so the color quenching was not considered in LSC measurement. And chemical quenching correction was mainly based on the salt content in the water sample. Under the condition of controlling the pH value of the solution, about 200 ml aqueous solution was concentrated to about 10 ml, and then 5 ml solution was transferred into the vial. Through spiked experiments, the average radiochemical recovery of the LSC method was 99.1%. The calculation results of activity concentrations of samples are shown in Table 7. From the SQP (E) values, acidity and solid content slightly affect the quenching. The absolute values of Z-Scores are all less than 2.0, in agreement with the reference values, which indicate the LSC method can provide satisfactory results.

**Table 7:** The results of gross alpha and gross beta activity concentrations for water samples from proficiency tests.

Sample No	Total solid content (mg L^−1^)	SQP(E)	Gross alpha activity concentration			Gross beta activity concentration		
			Reference value (Bq/L)*	Measured value (Bq/L)*	Z-Score	Reference value (Bq/L)*	Measured value (Bq/L)*	Z-Score
IS-2019	6.89 E + 02	796.3	(8.80 ± 0.30) E−01	1.04 ± 0.04	1.0	(6.20 ± 0.20) E−01	(6.92 ± 0.33) E−01	0.9
IS-2020	1.42 E + 03	795.4	(7.80 ± 0.40) E−01	(6.51 ± 0.55) E−01	− 1.2	(3.50 ± 0.20) E−01	(3.10 ± 0.24) E−01	− 0.8

*The expanded uncertainty corresponds to k = 1.

### Realistic applications

According to the currently reported literature, the countries that use the LSC method for routine monitoring mainly are Spain, Italy, Serbia, Finland, Mexico, and the United States. The types of water samples monitored include drinking water (well water, bottled purified water, and bottled mineral water), surface water, and groundwater^8–10,15,17,31–33^. However, the LSC method has not been approved in China, which is limited for laboratory research, and has not been applied for routine monitoring.

In this part, five representative types of water samples were selected, which were drinking water (DW), groundwater (GW), geothermal water (GT), seawater (SW), and wastewater (WW). By comparing the LSC method and the thick source method, the applicability of the LSC method for real sample monitoring was further investigated. The measurement results are shown in Table 8. Overall, the relative deviations of gross alpha and gross beta for ten samples were less than 15%, indicating that the two measurement methods were well comparable for these real water samples. For the cases of drinking water, groundwater, and geothermal water, the activity concentrations of gross α and gross β are less than 0.5 Bq/L and 1 Bq/L, respectively. For the case of seawater samples, the activity concentrations of gross α are less than 0.5 Bq/L, and the activity concentrations of gross β are 9–10 Bq/L. The main nuclide is ^40^K determined by gamma spectrometry. For the two wastewater samples, the activity concentrations of gross α and β are higher than the environmental levels, mainly containing ^241^Am and ^137^Cs (see Supplementary information, Table S1).

**Table 8:** The results of gross alpha and gross beta activity concentrations of different kinds of water samples determined using LSC and thick source methods.

Sample No	Total solid content (mg L^−1^)	LSC method^a^				Thick source method^a, b^			Deviation (%)^c^	
		Mass of the test sample (g)	SQP(E)	Gross alpha activity concentration (Bq L^−1^)	Gross beta activity concentration (Bq L^−1^)	Mass of the test sample (g)	Gross alpha activity concentration (Bq L^−1^)	Gross beta activity concentration (Bq L^−1^)	Gross alpha	Gross beta
DW-1	3.60 E + 02	2.02 E + 02	797.5	(4.16 ± 0.68) E−02	(1.21 ± 0.12) E−01	2.00 E + 03	(3.77 ± 0.85) E−02	(1.06 ± 0.11) E−01	10.3	14.2
DW-2	2.65 E + 02	2.02 E + 02	800.3	(2.55 ± 0.60) E−02	(1.11 ± 0.12) E−01	2.00 E + 03	(2.32 ± 0.68) E−02	(1.17 ± 0.12) E−01	9.9	− 5.1
GW-1	3.40 E + 02	2.02 E + 02	798.5	≤ MDC = 1.22 E−02	(7.92 ± 1.16) E−02	2.00 E + 03	≤ MDC = 1.81 E−02	(8.34 ± 1.21) E−02	–	− 5.0
GW-2	2.04 E + 02	2.03 E + 02	799.0	(2.45 ± 0.60) E−02	(3.77 ± 1.09) E−02	2.00 E + 03	(2.18 ± 0.52) E−02	(3.35 ± 0.98) E−02	12.4	12.5
GT-1	3.03 E + 02	2.04 E + 02	797.3	(1.88 ± 0.11) E−01	(5.82 ± 0.17) E−01	2.00 E + 03	(2.03 ± 0.14) E−01	(6.15 ± 0.23) E−01	− 7.4	− 5.4
GT-2	5.09 E + 02	2.04 E + 02	796.5	(1.84 ± 0.56) E−02	(5.11 ± 0.17) E−01	1.00 E + 03	≤ MDC = 3.87 E−02	(4.76 ± 0.15) E−01	–	7.4
SW-1	2.39 E + 04	5.03	793.4	≤ MDC = 4.97 E−01	9.91 ± 0.55	5.01 E + 01	≤ MDC = 1.41	9.34 ± 0.59	–	6.1
SW-2	3.22 E + 04	5.03	790.7	≤ MDC = 4.98 E−01	9.28 ± 0.55	5.00 E + 01	≤ MDC = 2.15	8.85 ± 0.51	–	4.9
WW-1	1.40 E + 04	5.05	796.9	(5.43 ± 2.21) E−01	(1.34 ± 0.01) E + 01	5.02 E + 01	(5.72 ± 2.90) E−01	(1.45 ± 0.03) E + 01	− 5.1	− 7.6
WW-2	9.81 E + 03	5.04	799.3	2.35 ± 0.30	6.27 ± 0.50	5.01 E + 01	2.16 ± 0.28	6.48 ± 0.57	8.8	− 3.2

^a^The expanded uncertainty corresponds to k = 1.

^b^The measurement time of samples and background are all 10 h.

^c^The deviation of the results of the LSC method relative to the results of the thick source method.

Compared the two measurement methods, for water samples with low salt content such as drinking water, groundwater, and geothermal water, the thick source method generally needs to heat and evaporate 1–2 L of water to obtain no less than 0.2 g of residue for measurement (the diameter of the planchet is 5 cm). At the same detection limit level, the LSC method only needs to heat and concentrate about 200 ml of water, and furthermore can avoid the troublesome sample laying process. Therefore, the pretreatment process of the LSC method is more rapid and efficient. For water samples with high salt content, such as seawater, the minimum detectable concentration of the LSC method is 3–4 times lower than that of the thick source method because of its high detection efficiency and solid capacity. For radioactive wastewater, the LSC method can be used for direct sampling and measurement, and the pretreatment process is simple, which can effectively avoid laboratory contamination and cross-contamination of samples.

For surface water and groundwater, M. Montana et al*.* compared the deviations of gross α measurement results of the LSC method and thick source method, and deeply analyzed the causes^34^. They thought that the gross α activity determined by the two methods was comparable for most of the studied water samples. This conclusion is consistent with the results of this paper. However, for some samples with high saline content or with very low gross α activity (close to the detection limit), it is observed that the deviations of the measurement results of the two methods increase (25–33%). In our work, no significant difference was observed for the two methods. We speculate that the possible reasons are the difference in the nature of the samples, sample treatment, and measurement conditions, or the insufficient number of samples we selected, which did not include all influencing factors, etc. In this aspect, more in-depth and detailed research is needed to do.

For real seawater and wastewater, there are few reports on measuring the gross α and gross β by the LSC method at present^20,35^. Our experimental results show that by controlling the acidity of the sample and the total amount of dissolved solids, the quenching level of the sample and the homogeneous state of the sample mixed with the scintillation liquid can be effectively controlled to ensure the reliability of LSC measurement.

## Conclusions

A method for simultaneous determination of gross alpha and gross beta activity concentrations in water by LSC was established. This method is suitable for measuring colorless environmental water samples (such as drinking water, groundwater, surface water, and geothermal water), seawater, and radioactive wastewater. The chemical quenching caused by dissolved salts in water is mainly considered in LSC measurement. A simulated salt solution is used as the chemical quenching agent to calibrate the LSC measurement method, which is validated by standard spiked samples. The measurement results of real samples show that the LSC measurement results are in good agreement with the measurement results of the thick source method. In addition, the LSC method has a simpler pretreatment process and a lower detection limit.

## Supplementary Information

Supplementary Information.

## Data Availability

The dataset used and analyzed during the current study available from the corresponding author on reasonable request.
