# Supplementary material for: Simultaneous determination of gross alpha/beta activities in water by liquid scintillation counting and its applications in the environmental monitoring
Source: Sci Rep. 2022 May 18;12:8281. doi: 10.1038/s41598-022-12245-x (PMC9117318; doi:10.1038/s41598-022-12245-x)
Supplement: Supplementary file 1 — Supplementary Information. [file 41598_2022_12245_MOESM1_ESM.docx]

**Supplementary Information for:**

**Simultaneous determination of gross alpha/beta activities in water by liquid scintillation counting and its applications in the environmental monitoring**

*Xiaoyun Li*, Shaolin Wang, Hailin Lou, Jingshun Pan, Qian Dong, Yifan Zheng, Ling Chen*

China Institute of Atomic Energy, Beijing 102413, China


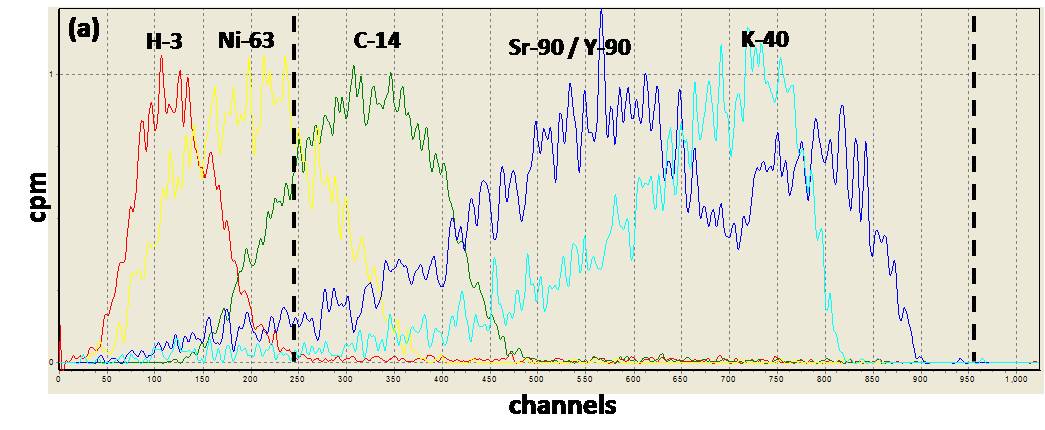


**Figure S1.** The LSC spectrums of representative beta nuclides (a) and alpha nuclides (b)

**Figure S2.** The comparison of the activity relative deviations of gross alpha (a) and beta (b) for pure spiked samples (PS), mixed spiked samples (MS), and mixed spiked samples containing soluble salts (MS+SS)

**Table S1.** The results of gamma spectrometry measurements for the seawater and wastewater samples

| Sample No. | Radionuclide activity concentration (Bq/L)^*^ | | |
| --- | --- | --- | --- |
|  | ^40^K | ^137^Cs | ^241^Am |
| SW-1 | 9.30 ± 0.45 | ≤ MDC = 2.32 E-01 | ≤ MDC = 2.60 E-01 |
| SW-2 | 9.89 ± 0.50 | ≤ MDC = 2.21 E-01 | ≤ MDC = 2.55 E-01 |
| WW-1 | ≤ MDC = 4.16 | (1.29 ± 0.08) E+01 | (6.08 ± 0.32) E-01 |
| WW-2 | ≤ MDC = 4.35 | 6.15 ± 0.38 | 2.09 ± 0.15 |

^*^ The expanded uncertainty corresponds to k = 1.
